# Genome-Wide Association Analysis for Chronic Superficial Keratitis in the Australian Racing Greyhound

**DOI:** 10.3390/genes13081328

**Published:** 2022-07-26

**Authors:** Steven Karamatic, Rebecca Goode, Niruba Bageswaran, Cali E. Willet, Georgina Samaha, Ray Ferguson, Hamutal Mazrier, Claire M. Wade

**Affiliations:** 1 Greyhound Racing Victoria, 46-50 Chetwynd Street, West Melbourne, VIC 3003, Australia; skaramatic@grv.org.au; 2 Greyhound Adoption Program, Greyhound Racing Victoria, 298 Goulburn Valley Hwy, Seymour, VIC 3660, Australia; bechall7@hotmail.com; 3 Faculty of Science, School of Life and Environmental Sciences, The University of Sydney, Camperdown, NSW 2006, Australia; niruba.kandasamy@sydney.edu.au; 4 Sydney Informatics Hub, The University of Sydney, Camperdown, NSW 2006, Australia; cali.willet@sydney.edu.au (C.E.W.); georgina.samaha@sydney.edu.au (G.S.); 5 Australian Greyhound Working and Sporting Dog Veterinarians, 1662 Dandenong Road, East Oakleigh, VIC 3166, Australia; rayferg@bigpond.net.au; 6 Faculty of Science, Sydney School of Veterinary Sciences, The University of Sydney, Camperdown, NSW 2006, Australia; hamutal.mazrier@sydney.edu.au

**Keywords:** greyhound, pannus, chronic superficial keratitis, Epidermal Growth Factor Receptor, *EGFR*

## Abstract

Chronic superficial keratitis (CSK) is a progressive inflammatory condition of the eye (cornea) that can cause discomfort and blindness. Differential disease risk across dog breeds strongly suggests that CSK has a genetic basis. In addition to genetic risk, the occurrence of CSK is exacerbated by exposure to ultraviolet light. Genome-wide association analysis considered 109 greyhounds, 70 with CSK and the remainder with normal phenotype at an age over four years. Three co-located variants on CFA18 near the 5′ region of the Epidermal Growth Factor Receptor (*EGFR*) gene were associated with genome-wide significance after multiple-test correction (BICF2P579527, CFA18: 6,068,508, p_raw_ = 1.77 × 10^−7^, p_genome_ = 0.017; BICF2P1310662, CFA18: 6,077,388, p_raw_ = 4.09 × 10^−7^, p_genome_ = 0.040; BICF2P160719, CFA18: 6,087,347, p_raw_ = 4.09 × 10^−7^, p_genome_ = 0.040) (canFam4)). Of the top 10 associated markers, eight were co-located with the significantly associated markers on CFA18. The associated haplotype on CFA18 is protective for the CSK condition. *EGFR* is known to play a role in corneal healing, where it initiates differentiation and proliferation of epithelial cells that in turn signal the involvement of stromal keratocytes to commence apoptosis. Further validation of the putative functional variants is required prior to their use in genetic testing for breeding programs.

## 1. Introduction

Chronic superficial keratitis (CSK) is a progressive inflammatory condition of the eye and cornea that if left untreated can result in complete blindness. Alternative names for the condition in the canine include pannus and Uberreiter’s syndrome [1]. Certain dog breeds express a higher prevalence of the disorder. For example, of 463 cases of the disorder observed in the Rocky Mountain region of the USA, more than 80% occurred in German shepherd dogs [1]. In the same study, greyhounds were also observed to express the disorder (1.3% of cases observed) but at an earlier age. The disorder has been reported to be present in 4% of retired racing greyhounds in the USA [2] but was unobserved in 2715 greyhounds treated in United Kingdom primary practices [3]. Variation in disease risk across breeds and disproportionate representation in greyhounds strongly suggests that CSK has a genetic basis [4]. Previous work has suggested that CSK risk may have dominant inheritance in the Australian Racing Greyhound [5]. Potential dominant inheritance is also supported by the appearance of the disorder in crossbreeds of German shepherd, which is another disproportionately affected breed [6]. In the German shepherd dog breed, candidate region analysis considering the Major Histocompatibility Complex has identified modest association between the disorder risk and locus homozygosity [7,8].

**Figure 1:**
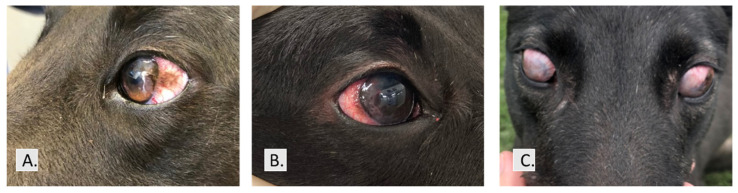
Ocular changes observed in Chronic Superficial Keratitis. (**A**) Early stages: raised, pigmented, and vascularized lesions commence development in the anterior corneal stroma. (**B**) Intermediate stage: vascularized regions advance with the disease progression. (**C**) Severe stage: granular tissue may eventually cover the cornea and the entire ocular surface.

Ocular changes associated with CSK typically arise as raised, pigmented, and vascularized lesions that commence as temporal/lateral lesions demonstrating development in the anterior corneal stroma (Figure 1A) and grow towards the center of the eye (Figure 1B). If untreated, granular tissue may eventually cover the cornea and the entire ocular surface (Figure 1C). It is suggested in the literature that younger affected dogs tend to express vascular lesions and severe manifestation of the disorder while dogs that are older on first diagnosis more often display pigmented lesions and milder disease [9]. The age of onset in dogs averages 4 years with most animals first diagnosed between 3 and 6 years [1]. Greyhounds as young as 1–2 years of age can show symptoms [10].

The corneal stroma is a transparent, viscoelastic tissue that resides posterior to a layer of non-keratinized, stratified epithelial cells (the corneal epithelium) that provides protection of the corneal stroma from superficial injury. The stroma is composed of multiple layers of collagen fibrils and glycosaminoglycan embedded with some keratocytes, originating from the neural crest [11]. The stroma lies in between the anterior cuboidal single layer of basement membrane (Bowman’s membrane) that controls interactions between the epithelial and stromal cells, and a monolayered endothelium to the posterior (Descemet’s membrane) that regulates corneal hydration. The keratocyte role is to maintain the fundamental integrity of the stroma as well as the regulation of stromal homeostasis of collagens and proteoglycans in the extra-cellular matrix (ECM) [11]. While most insults to the stroma are initiated by damage to the epithelium, this has not been observed to be the case in canine CSK [9]. The affected cells in CSK–stromal keratocytes arise from the neural crest [11].

During normal stromal wound healing, keratocytes first must undergo apoptosis. Some keratocytes also undergo necrosis, while keratocytes adjacent to epithelial injury are triggered to proliferate, migrate, and become activated to commence healing. In their activated state, the keratocytes are known as fibroblasts. The fibroblasts differentiate into myofibroblasts and together, these two cell types secrete a matrix scaffold that aids healing. Damaged epithelial and/or stromal cells produce chemokines that trigger an influx of inflammatory cells (an inflammatory response) to clear the necrotic and apoptotic debris. The inflammatory response in turn promotes the intrusion of blood vessels (neo-vascularization) into the stromal tissue to support the immune activation. Healing is slowly achieved by the resorption of abnormal ECM and the reverse differentiation or removal of the myofibroblast cells [12]. When this process fails transparency is not restored, resulting in corneal opacity. Decreased apoptosis of inflammatory cells can result in pathological neo-vascularization [12].

Canine CSK is regarded as incurable. However, the ocular defects in affected animals are non-infectious and typically respond to topical steroid-based treatments (e.g., prednisolone or dexamethasone), and therefore CSK is regarded as an autoimmune disorder. The autoimmune hypothesis has been supported by evidence of Major Histocompatibility Complex (MHC) involvement in German shepherd CSK [7,8] and gene expression patterns of cell infiltrates at the leading edge of the lesions [6]. MHC class II expression in the diseased eye cannot be conclusively regarded as the primary pathogenic cause of CSK. However, some authors believe it likely that MHC class II expression prolongs the inflammatory process in affected corneas and might provide an environment that favors the development of autoimmune reactions to antigens normally present in the canine cornea [13].

Evidence suggests that CSK risk is exacerbated by age, as well as exposure to environmental irritants such as smoke, ultra-violet light, and high altitude [10]. It is possible that an external agent may precipitate the primary lesion, triggering an alteration in the ocular immune response to self [14]. In humans, ocular keratitis may be precipitated by viruses [15]. Regardless of the initiating cause, it is very likely that a pathological immune response is responsible for the opacity and neovascularization that together characterize the CSK disorder.

## 2. Materials and Methods

Blood or saliva samples from 109 Australian racing greyhounds, comprising 39 greyhounds over four years old with no observable ocular abnormality upon veterinary inspection (controls) and 70 individuals CSK affected in one or both eyes (cases), were collected in Australia under University of Sydney Animal Ethics Committee (Approval numbers: 2015/902; 2018/1442 and 2021/2005) (Table S1). Genomic DNA was extracted from EDTA whole-blood samples using the PureLink Genomic DNA Mini Kit (Invitrogen, Waltham, MA, USA) following the manufacturer’s instructions. DNA quantity and quality was assessed using a NanoDrop2000 Spectrophotometer (Thermo Fisher Scientific, Waltham, MA, USA). DNA from blood spots applied to Whatman Flinders Technology Associates (FTA) cards was extracted by the genotyping provider (Neogen Inc., Lincoln, NE, USA). Performagene swab kit samples (DNAGenotek Inc., Kanata, ON, Canada) were purified with PG-L2P Performagene Purifier, and DNA was extracted following the protocol as described by the manufacturer.

DNA samples of 109 individuals were genotyped using the Illumina Canine 170K or 220K genotyping arrays (Neogen Inc., Lincoln, NE, USA). Three individuals were run on both 170K and 220K arrays to test for array calling concordance. Only the 220K arrays for the duplicated individuals were included in the main analyses. All duplicated individuals were CSK-affected. Genotyping markers (N = 1500) exhibiting the most disparate calls between the animals genotyped on both array types were excluded.

Illumina HiSeq whole-genome sequences were generated for three animals: one CSK affected (ID 1455); one control (ID 3181); and one with unknown CSK sequenced for a different study (ID 3343) using 100 base paired-end TruSeq libraries (Ramaciotti Centre for Functional Genomics, Kensington, NSW, Australia) at 30× coverage and with a 250 base pair insert size. Two of these individuals were included in the array-based genotyping. The restriction of the association analysis to animals phenotyped for CSK meant that ID 3343 was not included in the genome-wide association analysis.

Standard case–control genome-wide association analysis (GWAS) was conducted using Plink [16]. Data were quality filtered for marker call rate (--geno 0.1) and minor allele frequency (--maf 0.05). Individuals with more than 30% missing calls were excluded. This value allowed for animals with 170K array data (by default 23% missing calls relative to animals with 220K arrays) to remain in the analysis. Case–control analysis included only the animals with observed CSK phenotype (70 cases and 39 controls). Variants were regarded as genome-wide significant if they exhibited p_genome_ < 0.05. Multiple test correction was based on the Bonferroni statistic from applying the adjusted flag (--adjusted) in the PLINK association analysis.

Genotypes for individual chromosomes exhibiting genome-wide significance were extracted and analyzed separately using Haploview [17] to detect haplotype-based association. The same quality filtering was applied to the refined set of markers.

Fastq files prepared from paired-end sequencing reads of whole-genome sequenced animals were aligned with the UU_Cfam_GSD_1.0/canFam4 reference genome (canFam4) [18] using the Burrows Wheeler Aligner [19] and variants were called using standard pipelines using samtools, mpileup, and bcftools [20] as described previously [21].

Variant calls were captured for regions of association defined as 500Kb before and after genome-wide associated markers. Putative variant function for the linked variants was assessed after uploading variant call files (VCF) generated for chromosomal regions of interest from the whole-genome sequenced greyhounds as a custom track to the University of Santa Cruz genome browser (canFam4). The annotations used for comparison were the Uppsala University GSD1.0 gene annotations track (UU-GSD1) [18] and the “NCBI RefSeq genes, curated and predicted” available via the Variant Annotation Integrator (VAI) tool [22]. Analysis considered variants in transcribed portions of protein coding genes and exon splice sites.

Local alignments were assessed via the Integrated Genomics Viewer (IGV) to detect potential novel repeat element insertion and structural variants in associated regions [23]. Wider frequency of putative functional variants was assessed using two public canine genotyping resource archives [24,25]. To assess variant frequency in wider dog populations, the UCSC Genome browser “Liftover” tool was used to locate features between the canFam4 and canFam3.1 reference genomes.

## 3. Results

### 3.1. Genome-Wide Association Analysis

**Figure 2:**
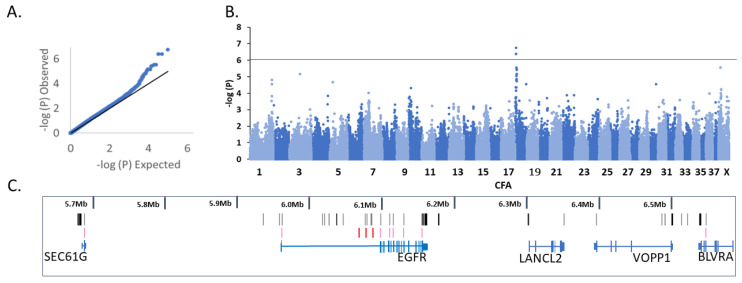
Genome-wide association analysis for Chronic Superficial Keratitis in Australian racing greyhounds (Cases 66, Controls 39). (**A**) Quantile-quantile plot. (**B**) Manhattan plot (Lamda = 1.14). (**C**) Genes in the association region showing the three genome-wide significant markers (BICF2P579527, CFA18: 6,068,508, p_raw_ = 1.77 × 10^−7^, p_genome_ = 0.017; BICF2P1310662, CFA18: 6,077,388, p_raw_ = 4.09 × 10^−7^, p_genome_ = 0.040; BICF2P160719, CFA18: 6,087,347, p_raw_ = 4.09 × 10^−7^, p_genome_ = 0.040)) as red bars. Putative functional variants in exons and splice-sites are shown as pink bars. All possible functional variants including variants in UTRs and non-coding RNAs shown as black bars. All positions refer to canFam4.

From the primary association analysis on unstratified, phenotyped greyhounds, 66 cases and 39 controls passed quality filtering. Four dogs were lost due to poor genotyping rate. The GWAS included 98,271 markers (54,422 were removed due to missing calls, 66,435 variants removed due to minor allele frequency, and 226 variants removed due to Hardy–Weinberg exact test). The median Chi-squared value was 1.14 (Figure 2A,B).

From the primary analysis, three co-located variants (BICF2P579527, CFA18: 6,068,508, p_raw_ = 1.77 × 10^−7^, p_genome_ = 0.017; BICF2P1310662, CFA18: 6,077,388, p_raw_ = 4.09 × 10^−7^, p_genome_= 0.040; BICF2P160719, CFA18: 6,087,347, p_raw_ = 4.09 × 10^−7^, p_genome_ = 0.040 (canFam4)) exhibited genome-wide significance after multiple-test correction. The three variants with genome-wide significance were tightly clustered near the first exon of the Epidermal Growth Factor Receptor (*EGFR*) gene (Figure 2C). Of the top 10 associated markers, eight were co-located within five megabases (Mb) of the three most significantly associated markers. Other markers represented in the top 10 came from chromosomes CFX: 8,930,555 and CFA3:47,190,469 (Table S2).

### 3.2. Variant Analysis

#### 3.2.1. CFA18

The defined region of association spanned CFA18:5,568,508–6,587,347 (canFam4). This region contains five annotated protein coding genes: Protein Transport Protein Sec61 Subunit γ (*SEC61G*), *EGFR*, LanC-Like Protein 2 (*LANCL2*), Vesicular, Overexpressed In Cancer, Prosurvival Protein 1 (*VOPP1*), and Biliverdin-IX α-Reductase (*BLVRA*). The primary association signal on CFA18 was located close to a plausible positional candidate gene: *EGFR*. From the array-based genotyping, our expectation was that the control animal ID 3181 would harbor the associated protective haplotype as a heterozygote in the region of association. By array genotypes, affected individual 1455 and the unknown individual 3343 would share the homozygosity for the risk haplotype.

The greyhound whole-genome sequence alignments failed in the region of the first coding exons of *EGFR* and *VOPP1* identified using the canFam4 reference assembly and the UU-GSD1.0 gene annotations. The two phenotyped individuals demonstrated inferior alignment to the individual 3343 that was sequenced with a later technology. The sequencing/alignment failure was presumed to be caused by locally high genomic G-C content.

Variant calling from the three greyhounds with whole genome sequencing identified 2863 variants in the significant association region (Table S3). Potential functional variants are shown in Table S4. Variants potentially affecting protein coding in the known genes (*SEC61G*, *EGFR*, *LANCL2*, *VOPP1,* and *BLVRA*) are shown in Table 1. The VAI identified 35 unique potentially functional variants based on the filtering criteria: 8 3′UTR, 1 5′UTR, 1 missense, 20 non-coding-transcript exon variants, 1 splice-region variant, and 4 synonymous coding variants (Table S4). In addition, a further 79 potentially functional variants were added by considering the UU-GSD1 functional annotations (43 3′-UTR, 8 5′-UTR, 1 stop codon variant, 1 splice-acceptor site variant, 1 synonymous coding variant, and a further 25 variants coded in long non-coding RNA or putative protein coding transcripts) (Table S4). The three genome-wide significant array markers are included in Table 1 and Table S4 for reference. Significant array markers (red bars), exonic variants (pink bars), and splice variants (pink bars) are included in Figure 2C.

Special attention was paid to the region in the 5′ region of EGFR since this was located close to the association signal. A splice site mutation affecting intron 2 of EGFR, NM_201283, CFA18:5,961,614 rs851737129G > C was observed. At this variant, the affected individual is homozygous for the reference allele that retains the “GG” splice-acceptor while the control and un-phenotyped individuals are heterozygous for the alternate allele altering the splice acceptor site to “CG”. One non-synonymous variant CFA18:6,110,158G > A NM_201283p.255R > Q in EGFR (new), predicted to be neutral by PROVEAN [26], was present as a heterozygote only in control ID 3181 (Table 1). One variant potentially affecting the stop codon for human transcript NM_001346897.2 CFA18: 6,154,817 rs397512405T > C was shown upon closer inspection to be likely not functional in the dog (reference codon sequence TAT, new sequence TAC). A second splice-region variant in *EGFR* (CFA18:6,115,276) and the potential stop-codon variant were heterozygous in both the case and control sequenced animals. The protective haplotype identified in the GWAS is present only in ID3181 as a heterozygote. This haplotype likely affects *EGFR* activity, either through splicing, or in combination with other variants that such as the non-synonymous variant. The presence of multiple potential functional variants in the *EGFR* gene may contribute to the complexity of the inheritance.

#### 3.2.2. CFX

While no markers on CFX attained genome-wide significance, the imperfect association with the CFA18 locus invited us to consider other signals from the analysis. The fifth most associated marker in the analysis was located on the X chromosome CFX: 8,930,555 (canFam4) (variant BICF2G630538106). The association interval was defined as CFX:8,430,655–9,430,555 (canFam4) by allowing 500 Kb of linkage-disequilibrium with the most associated marker. The CFX region VCF for three greyhounds with whole genome sequence is provided in Table S5. The region includes five protein coding genes: FERM And PDZ Domain Containing 4 (*FRMPD4*), Phosphoribosyl Pyrophosphate Synthetase 2 (*PRPS2*), Toll-like Receptor 7 (*TLR7*), Toll-like Receptor 8 (*TLR8*), and Thymosin β 4 X-Linked (*TMSB4X*), including at least two genes that plausibly may affect CSK risk: the toll-like receptor genes (*TLR7* and *TLR8*). Both genes have been shown to affect autoimmunity in mouse and human models including ocular allergic and autoimmune diseases [27]. Variant annotation from the three sequenced greyhounds identified missense variants in *TLR8* (three missense variants). This region accounted for eight variants among the 50 most associated array variants, including four in the defined region of association. Other potentially functional variants were observed in the association region (Table S6). Strong divergence in the *TLR8* gene between the affected ID 1455 and unaffected ID 3181 sequenced dogs was observed. All three observed missense variants CFX:g. 9,365,039 C > A, XM_005641119:.p.104P > T (no existing variant identifier), rs24607342, CFX:g.9,366,054G > A, XM_005641119.3:p.442G > S, and BICF2G630537785, CFX:g.9,366,787G > A, XM_005641119.3:p.686R > H, demonstrated divergence between the case and control sequenced animals. All observed missense variants in *TLR8* are individually predicted to be benign/tolerated [26], though inherited as a haplotype they may affect the gene efficacy or specificity.

#### 3.2.3. CFA3

On CFA3, CFA3:47,190,469 (canFam4) (TIGRP2P43485_rs9072900) is intergenic, lying in-between and five-prime to two genes: multiple C2 and transmembrane domain-containing protein 2 (MCTP2) (CFA3:46,324,673–46,460,165) and repulsive guidance molecule A precursor (RGMA) (CFA3:47,534,393–47,578,334). The associated marker was alone at this locus among the top 50 markers in the analysis. Genes in the vicinity have not been connected with corneal function and so this locus was excluded from further consideration.

### 3.3. Haplotype Risk

Risk of clinical disease for haplotypes on CFA18 and the most associated marker on CFX are shown in Table 2. Perfect association with CSK was not observed at either locus. Animals homozygous for the risk haplotype on CFA18 had 76% risk of exhibiting CSK. On CFX, equal numbers of male and female cases were observed and animals exhibiting only the risk allele had 71% risk of CSK.

## 4. Discussion

While the literature suggests that younger affected dogs tend to express vascular lesions and severe manifestation of the CSK disorder, routine screening that resulted in the cases used in this study detected predominantly milder lesions indicative of early signs of the disease. During observed management, the lesions were responding well to initial treatments with reduced progression of lesions across the cornea.

Associated markers on CFA18 were located near a gene with potential to impact the observed phenotype. *EGFR* is known to play a role in corneal healing, where it initiates differentiation and proliferation of epithelial cells that in turn signal the involvement of stromal keratocytes to commence apoptosis [12]. The CSK phenotype is not commonly understood to involve epithelial injury, as fluorescein staining of CSK affected eyes does not typically indicate the involvement of the epithelial ulceration in the disorder [9]. Despite no obvious epithelial injury, CSK affected eyes demonstrate mild to severe hyperplasia of the corneal epithelium and thickening of the epithelial basement membrane [28,29].

Throughout the CFA18 region of association with the CSK trait, we observed a considerable amount of genetic diversity among the three sequenced greyhounds that made phasing of haplotypes challenging. Poor alignment in the vicinity of the EGFR first exon hampered genotyping and validation in this region, which was the site of the strongest association signal. Two variants demonstrating the greatest concordance among those assessed for function in the sequenced greyhounds, were a splicing mutation (rs851737129G > C) affecting exon 2 of *EGFR* affecting the splicing of K28 with K29 and V30 in the protein (NM_201283), and a missense mutation in *EGFR*: CFA18: 6,110,158G > A *EGFR*p.255R > Q (new). At the rs851737129G > C variant, the affected sequenced dog had the wild-type allele while the control dog 3181 was heterozygous for the splicing variant that is rare in the broader canine population. Dampening the effect of *EGFR* may reduce the proliferation of epithelial cells. If the CSK phenotype is a result of an overactive wound healing process, then restricting the activity of this process might result in a less severe disease course.

Markers on CFX did not reach genome-wide significance but showed a sustained regional association that was the second most associated in the study. However, the X-chromosome locus includes three observed coding mutations in *TLR8* and strong divergence in the sequence of *TLR8* between the control (ID 3181) and affected (ID 1455) sequenced greyhounds. Toll-like receptor (TLR) genes are important components of the innate immune system, and their disruption is known to trigger autoimmune responses in other species [30]. In certain specific cellular conditions, TLR genes may be inappropriately activated by self-components of the cell, and this activation may lead to sterile inflammation and autoimmunity. TLR genes occur in classes that respond to various pathogenic characteristics. *TLR8* is a part of a group of TLR genes that responds to nucleic acids, and particularly single-stranded RNAs (for example, viruses). In addition to a non-specific connection with autoimmune responses, TLR genes have been specifically connected with ocular keratitis in humans [15].

Typically, recessive X-chromosome disease variants generate a sex imbalance in the affected individuals, such that males are over-represented in the disease cohort. In our analysis, the balance between male and females in the affected cohort was very even (34 male and 36 female). Dominant modes of inheritance for X-chromosome traits can occur and, in this situation, females and males may both be affected equally. The defining feature of X-linked dominant inheritance is that males cannot transmit the trait to their sons. If the *TLR8* signal is affecting CSK autoimmunity, it is likely that dominant inheritance is affecting the trait. Breeding of affected individuals is not encouraged by the breed community, although if the breeding occurred before the disorder became apparent (since age of onset is typically greater than four years) it might be possible that some parental animals could be affected. The limited pedigree information that we have for this cohort does not offer further information to answer this question as we have no parental animals recorded as affected in the pedigrees accessed. Currently, while the connection with autoimmunity is enticing, the direct connection between variants in *TLR8* and CSK is unproven.

## 5. Conclusions

Genome-wide analysis in the Australian racing greyhound has identified a region on CFA18 that is significantly associated with risk of CSK. The associated haplotype identified by the analysis appears to reduce the risk of disease. The identified haplotype does not perfectly explain the disease in the Australian dogs and this suggests that other loci likely affect the condition in this breed. The characteristics of the CSK phenotype show strong concordance with the corneal wound healing process that is plausibly affected by the gene *EGFR*. A second association signal in the analysis that failed to reach genome-wide significance identified a toll-like receptor locus on the X chromosome. Toll-like receptor genes have been shown to control autoimmunity in a range of species. While both of the main loci identified in this analysis might play a role in the CSK disease, we propose that at this time, the use of the CFA18 and CFX loci for genetic testing is premature. Genetic research on this phenotype is continuing.

## Figures and Tables

**Table 1:** Characteristics of putative protein-coding variants and array markers on CFA18.

Position on CFA18 (bp)	5,961,614	6,068,508	6,077,388	6,087,347	6,110,158	6,115,276	6,154,817
Variant identifier	rs851737129	rs22613273	rs22642459	rs22653533	.	.	rs397512405
Location	EGFR	ARRAY	ARRAY	ARRAY	EGFR	EGFR	EGFR
Feature	splice_region_variant	intronic	intronic	intronic	missense_variant	splice_region_variant	potential stop codon
Reference allele ^a^	G	A	C	T	G	A	T
Alternate allele (s)	C	G	T	C	A	T	C
USCF1455 (case)	G G	G G	T T	C C	G G	T A	C T
USCF3181 (control)	C G	A G	C T	T C	A G	T A	C T
Source of annotation	UU-GSD1 ^a^	ARRAY ^c^	ARRAY	ARRAY	VAI ^b^	VAI	UU-GSD1
MAF_722_^d^	0.552	0.397	0.432	0.383	0.0009158	0.063	0.132
MAF_590_^e^	0.491	0.479	0.486	0.476	-	0.036	0.074

^a^ University of Uppsala GSD1.0/canFam4. ^b^ VARIANT ANNOTATION INTEGRATOR (VAI) Hinricks et al. (2016). ^c^ Illumina Canine Genotyping Array (170K and 220K). ^d^ Minor Allele Frequency (MAF) Plassais et al. (2019). ^e^ MAF Jagannathan et al. (2019).

**Table 2:** Haplotype risk for the CFA18- and CFX-associated markers.

		Case	Control	Penetrance
CFA18 Haplotype ^a^	Homozygous GAG (Risk18)	65	21	0.76
	Heterozygous	3	16	0.16
	Homozygous AGA (Low-risk18)	0	1	0.00
	Uncalled	2	2	
CFX:BICF2G630538106 ^b^	G G (RiskX)(Female) G (Male)	63	26	0.71
	A G (Female only)	3	5	0.38
	AA (Low-riskX) (Female) A (Male)	2	8	0.20
	Uncalled	2	0	
Interaction	Risk18/RiskX	61	15	0.80
	Risk18/Low-riskX	2	4	0.33
	Low-risk18/RiskX	0	0	-
	Low-risk18/Low-riskX	0	1	0.00
	Other	7	19	0.27

^a^ BICF2P579527, CFA18: 6,068,508, p_raw_ = 1.77 × 10^−7^, p_genome_ = 0.017; BICF2P1310662, CFA18: 6,077,388, p_raw_ = 4.09 × 10^−7^, p_genome_ = 0.040; BICF2P160719, CFA18: 6,087,347, p_raw_ = 4.09 × 10^−7^, p_genome_ = 0.040. ^b^ BICF2G630538106, CFAX:8,961,843, p_raw_ = 2.85 × 10^−6^, p_genome_ = 0.28.

## Data Availability

Whole genome sequencing data connected with this project are available via the European Nucleotide Archive https://www.ebi.ac.uk/ena/browser/view/PRJEB53922 (22 July 2022).

## Supplementary Materials

The following supporting information can be downloaded at: https://www.mdpi.com/article/10.3390/genes13081328/s1. Table S1. Characteristics of greyhound samples used in the current analysis. Table S2. Ten most associated markers by genome-wide association analysis. Table S3. Variant call file for associated locus on CFA18 including genotypes from three Australian racing greyhounds (1455-Case; 3181-Control; 3343-Unphenotyped). Table S4. Characteristics of 117 putative functional variants and array markers on CFA18 showing genotypes in three Australian racing greyhounds. Table S5. Variant call file for associated locus on CFX including genotypes from three Australian racing greyhounds (1455-Case, 3181-Control, 3343-Unphenotyped). Table S6. Putative functional variants in region of most associated marker on CFX-CFX:8,430,655–9,430,555 (canFam4).
